# Association of SARS-CoV-2 Infection with Neurological Symptoms and Neuroimaging Manifestations in the Pediatric Population: A Systematic Review

**DOI:** 10.21203/rs.3.rs-2653722/v1

**Published:** 2023-03-09

**Authors:** Angela T.H. Kwan, Khaled Al-Kassimi, Jacob S. Portnoff, Marija Tesla, Mehrshad Hanafimosalman, Nima Gharibi, Tiffany Ni, Davaine J.N. Sonfack, Julia Martyniuk, Saman Arfaie, Mohammad Sadegh Mashayekhi, Mohammad Mofatteh, Richie Jeremian, Luis Rafael Moscote-Salazar, Ángel Lee, Muhammad Youshay Jawad, Ziji Guo, Felicia Ceban, Kayla M. Teopiz, Rodrigo B. Mansur, Roger Ho, Joshua D. Rosenblat, Bing Cao, Taeho Greg Rhee, Roger S. McIntyre

**Affiliations:** University of Ottawa; American University in the Emirates; The University of Queensland; University of Toronto; McGill University; Saint James School of Medicine; University of Toronto; Laval University; University of Toronto; McGill University; University of British Columbia; Queen’s University Belfast; McGill University; University of Cartagena; Hospital Ángeles del Pedregal; Brain and Cognition Discovery Foundation; Brain and Cognition Discovery Foundation; Brain and Cognition Discovery Foundation; Brain and Cognition Discovery Foundation; University of Toronto; Yong Loo Lin School of Medicine; University of Toronto; Southwest University; Yale University; University of Toronto

**Keywords:** COVID-19, cognition fatigue, MIS-C, neurological symptoms, neuroimaging manifestations, pediatrics, systematic review

## Abstract

**Background:**

Neurological manifestations have been widely reported in adults with COVID-19, yet the extent of involvement among the pediatric population is currently poorly characterized. The objective of our systematic review is to evaluate the association of SARS-CoV-2 infection with neurological symptoms and neuroimaging manifestations in the pediatric population.

**Methods:**

A literature search of Cochrane Library; EBSCO CINAHL; Global Index Medicus; OVID AMED, Embase, Medline, PsychINFO; and Scopus was conducted in accordance with the Peer Review of Electronic Search Strategies form (October 1, 2019 to March 15, 2022). Studies were included if they reported (1) COVID-19-associated neurological symptoms and neuroimaging manifestations in individuals aged < 18 years with a confirmed, first SARS-CoV-2 infection and were (2) peer-reviewed. Full-text reviews of 222 retrieved articles were performed, along with subsequent reference searches.

**Results:**

A total of 843 nonduplicate records were retrieved. Of the 19 identified studies, there were ten retrospective observational studies, seven case series, one case report, and one prospective cohort study. A total of 6,985 individuals were included, where 12.8% of hospitalized patients experienced neurocognitive impairments: MIS-C (24.2%), neuroinflammation (10.1%), and encephalopathy (8.1%) were the most common disorders; headaches (16.8%) and seizures (3.8%) were the most common symptoms. Based on pediatric-specific cohorts, children experienced more drowsiness (7.3% vs. 1.3%) and muscle weakness (7.3% vs. 6.3%) as opposed to adolescents. Agitation or irritability was observed more in children (7.3%) than infants (1.3%).

**Conclusion:**

Our findings revealed a high prevalence of immune-mediated patterns of disease among COVID-19 positive pediatric patients with neurocognitive abnormalities.

## Introduction

Coronaviruses usually cause respiratory illness, but the highly pathogenic SARS-CoV-2 virus—responsible for COVID-19—is also associated with peripheral and central nervous system (CNS) disorders (1). In adults, its clinical presentation is heterogenous, ranging from asymptomatic or mild respiratory tract symptoms to severe pneumonia with acute respiratory distress syndrome (ARDS) and multiorgan dysfunction (2). During the acute stage, severe neurological and psychiatric complications may also manifest, where development of cytokine storm and thrombogenic reactions result in a high incidence of ischemic stroke and intracerebral hemorrhage (3, 4). Conversely, the pediatric population is much less susceptible to serious SARS-CoV-2 infection; only 0.4% of severe cases involved children (3, 5). A large percentage of children are either asymptomatic or pre-symptomatic. They generally present milder symptoms of COVID-19 and are rarely hospitalized due to a lower risk of exposure, higher levels of antibodies against viruses, and maturing angiotensin-converting enzyme 2 (ACE2) (5, 6). However, as the pandemic progressed, reports of immune-mediated patterns of disease began to emerge; it became evident that other systemic symptoms could develop alongside respiratory involvement (3).

Recently, there is growing evidence suggesting the development of a systemic secondary inflammatory response among children related to severe acute SARS-CoV-2 infection, termed Multisystem Inflammatory Syndrome in Children (MIS-C)—also known as Pediatric Inflammatory Multisystem Syndrome Temporally Associated with COVID-19 (PIMS-TS) (3). While the majority of adults experiencing severe COVID-19 infection were hospitalized for respiratory complications such as ARDS, children presented with MIS-C (7). MIS-C is a relatively rare (2 per 100,000), post-infectious illness that is associated with a high prevalence of neurological symptoms (34%) and abnormal neuroimaging findings (3). But, the extent of neurocognitive complications of MIS-C still remains poorly characterized (8). Moreover, four other major syndromes were observed to be associated with COVID-19 infection in children – namely, bronchopulmonary syndrome, gastrointestinal syndrome, fever without a source, and mild syndrome (5).

Due to isolated reporting, it is difficult to discern the frequency and patterns of COVID-19-related CNS disorders (3). To address this challenge, the systematic review explores the association of SARS-CoV-2 infection with neurological symptoms and neuroimaging manifestations across the pediatric population (< 18 years old).

## Methods

This systematic review was conducted according to the Preferred Reporting Items for Systematic Reviews and Meta-Analysis (PRISMA) guidelines (9).

### Search Strategy

Peer-reviewed literature was identified using a comprehensive search strategy designed and executed by the medical librarian (JM), which was reported based on the PRISMA search extension checklist developed by Rethlefsen et al. (2021) (eTable 1). The search scope was enhanced and errors were minimized through consultation with the review team, and the OVID Medline search was peer reviewed by a research services librarian in accordance with the Peer Review of Electronic Search Strategies (PRESS) form. The full search strategies, grey literature search, and PRESS form can all be located here: https://doi.org/10.5683/SP3/VAPXLL.

Subject headings and textwords related to the following concepts were included in the search: ‘brain imaging,’ ‘brain structure/function,’ ‘covid,’ and ‘pediatrics.’ The search strategy for the concept of COVID-19 was adapted from two published search strategies while the concept of pediatrics was adapted from four published search strategies (10–14).

The search strategy was developed and finalized in OVID Medline and then translated to the Cochrane Library, EBSCO CINAHL, Global Index Medicus, OVID AMED, OVID Embase, OVID PsychINFO, and Scopus. The search was filtered to human participants. No language restrictions were applied. A date limit was implemented to include articles published since 2019. The searches were run on December 22, 2021 and – prior to final analysis – rerun on March 15, 2022 in order to incorporate papers published since the review began. The search results were exported into Endnote and subsequently imported into Covidence for two rounds of deduplication prior to screening.

Relevant grey literature was identified by hand searching grey literature databases and catalogs, including BioRxiv, ClinicalTrials.gov, ISRCTN Registry, MedRxiv, and the World Health Organization International Trials Registry Platform. Hand searching of the reference lists of included articles and previous reviews was also performed to identify studies that may have been missed.

### Screening

Two reviewers (ATHK and JSP) independently conducted title and abstract screening on the retrieved articles based on the predefined inclusion/exclusion criteria. Full-text screening was then independently conducted by two reviewers (ATHK and JSP) to reassess their eligibility for inclusion in the data extraction stage. Any subsequent discrepancies were resolved by discussion or by cross-referencing a third reviewer (KAK). Additionally, a manual search was performed by two reviewers (ATHK and JSP) on selected references since September 2021 to ensure that additional appropriate articles published after conducting the search strategy were screened for eligibility as well.

### Eligibility Criteria

Our inclusion criteria included the following: original, peer-reviewed research articles (from “2019 October” to present); all studies (e.g., case reports, case-control studies, cohort studies, observational studies, pilot studies, preprints, randomized controlled trials, registered clinical trials, and other study designs) that examine the impact of SARS-CoV-2 infection on brain structure and function in the pediatric population; individuals under 18 years of age; patients with a confirmed, first SARS-CoV-2 infection; scanned for structural and functional changes using brain CT, MRI, PET scan, or other modalities; studies reporting sufficient details on neuroimaging findings; cohort studies and case-series without group control to aid with additional data; and available full-text article.

Our exclusion criteria included the following: abstracts, dissertations, editorials, guidelines, perspective papers, or review articles; unconfirmed cases of COVID-19; patients with pre-existing neurological comorbidities; studies conducted only on animals; and studies lacking explicit reporting or inclusion of sufficient details on neuroimaging findings and COVID-19 patient performances based on standard cognitive assessments.

### Data Extraction

A standardized data extraction form was developed on a Microsoft Excel spreadsheet (version 2016; Microsoft, Redmond, WA, USA) and included the following headings: article title, first author and publication year, country of study, study design and methodology, demographic information: age (including mean and median) and gender, sample size (COVID-19 positive children), COVID-19 confirmation, COVID-19 severity stage, affected sample size (COVID-19 positive children with neurological symptoms and/or neuroimaging manifestations), and neurological complications (type of disorder and time of appearance).

### Assessment of Bias and Methodological Quality

Each selected article underwent risk of bias assessment according to the study design (Table 1). The JBI Critical Appraisal Checklist for Case Series, Cohort Studies, and Cross-sectional Studies were used based on the following domains: presence of attrition bias (number of withdrawals and drop-outs), detection bias, performance bias, reporting bias, selection bias, and ‘others’ (15, 16). Each study was awarded one (“yes”) or zero (“no”) points on the JBI checklist; total points along with maximum attainable points were included. Two reviewers (MYJ and ZG) independently performed this task before coming to a mutual conclusion. All discrepancies were resolved through discussion and arbitration by a third author (ATHK).

## Results

A total of 19 studies were included in this systematic review, which examined the association between SARS-CoV-2 infection and neurological symptoms and atypical neuroimaging manifestations in the pediatric population. Overall, it was found that 12.8% (*n* = 892 of 6,985) of hospitalized patients experienced neurocognitive impairments. By order of decreasing prevalence, it is noteworthy that MIS-C (*n* = 216 of 892, 24.2%) was the most common disorder, followed by neuroinflammation (*n* = 90 of 892, 10.1%)—which includes acute disseminated encephalomyelitis (ADEM) or encephalitis or myelitis or meningism or meningitis or meningoencephalitis—and encephalopathy (*n* = 72 of 892, 8.1%). Moreover, headaches (*n* = 150 of 892, 16.8%) and seizures (*n* = 34 of 892, 3.8%) were among the most commonly reported neurological symptoms. When examining symptom prevalence by age group, drowsiness was found to be more prevalent among children (*n* = 8 of 78, 7.3%) as opposed to adolescents (*n* = 1 of 52, 1.3%). More children also experienced muscle weakness (*n* = 8 of 78,7.3%) as compared to adolescents (*n* = 5 of 52, 6.3%). Agitation or irritability was observed more in children (*n* = 8 of 78, 7.3%) than infants (*n* = 1 of 6, 1.3%). These estimates along with the primary findings of their associated articles are summarized in Table 2.

### Systematic Search Results and Study Characteristics

**Figure 1:**
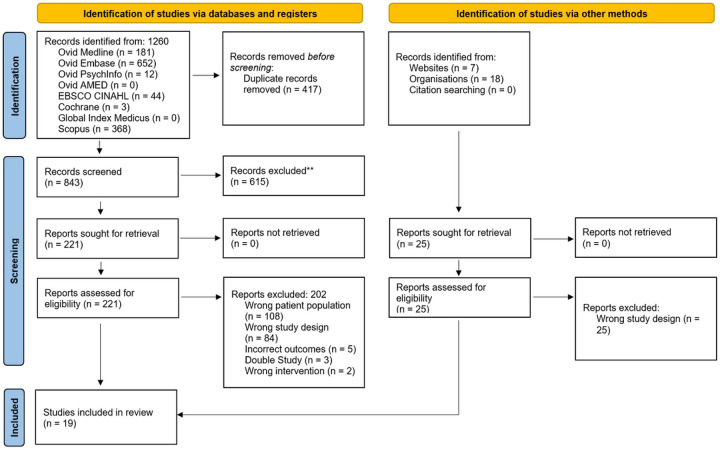
Flow diagram for the systematic review based on the PRISMA criteria. From: Page MJ, McKenzie JE, Bossuyt PM, Boutron I, Hoffmann TC, Mulrow CD, et al. The PRISMA 2020 statement: an updated guideline for reporting systematic reviews. BMJ 2021;372:n71. doi: 10.1136/bmj.n71.

The electronic databases were systematically searched at two time points, 12/22/2021 and 05/15/2022, due to the ongoing publication of COVID-19 studies and a small number of published studies on pediatric cognition. There were 1260 studies identified in the databases – where 417 were removed as duplicates. The remaining 843 were screened by title and abstract based on the inclusion and exclusion criteria—yielding 221 eligible studies for full-text assessment. Of the 221 papers, only 19 met the inclusion criteria and 202 were excluded for the following reasons: wrong patient population (*n* = 108), wrong study design (*n* = 84), incorrect outcomes (*n* = 5), double study (*n* = 3), and wrong intervention (*n* = 2) (Fig. 1).

Of the 19 studies, ten were retrospective observational studies, seven were case series, one was a case report, and one was a prospective cohort study. Details of study selection are illustrated in Fig. 1.

Studies were published between February 2020 and May 2021 and included data from hospital centers from four continents and 16 different countries—including Argentina, Brazil, Chile, France, India, Iran, Italy, Mexico, Peru, Romania, Saudi Arabia, Spain, Switzerland, Turkey, United Kingdom, and the United States. Four studies analyzed data from Turkey; three from France, United Kingdom, and United States; two from Brazil, India, Peru, and Saudi Arabia; and one from Argentina, Chile, Italy, Iran, Mexico, Romania, Spain, and Switzerland.

### Quality Assessment Results

The component studies in our systematic review consisted of case reports/series, cross-sectional studies, and cohort studies. Overall, studies were of moderate quality with some concerns across some key domains. Since JBI does not provide domain-based scoring, a numeric value was assigned to the study delineating its overall quality. Detailed information regarding the study, the tool applied, and obtained and maximum scores are presented in Table 1.

### Clinical Presentation: Neurocognitive Impairments

Study characteristics and primary findings are reported in Table 2. The sample size ranged from 3 to 3,694, with a total of *n* = 6,985 pediatric patients (< 18 years old). All of these individuals had a confirmed COVID-19 diagnosis either by QRT-PCR (nasopharyngeal swab) or positive SARS-CoV-2 IgG (serum). Among pediatric studies that reported age of the population (*n* = 136 patients), we determined that 4.4% were infants (≤ 1 years old, *n* = 6 of 136), 57.4% were children (> 1 to ≤ 12 years old, *n* = 78 of 136), and 38.2% were adolescents (> 12 to < 18 years old, *n* = 52 of 136).

It was found that 12.8% (*n* = 892 of 6,985) of all hospitalized patients had neurocognitive symptoms or abnormal neuroimaging findings. Females (*n* = 113 of 252) consisted of 44.8% of individuals with neurological involvement. The following studies did not report sex of the patients: Abdel-Mannan et al., 2020, Dilber et al., 2021, Fenlon III et al., 2021, LaRovere et al., 2021, Sa et al., 2021, and Ucan et al., 2022.

The range of neurological symptoms associated with COVID-19 was heterogeneous and included the following: headaches (*n* = 150 of 892, 16.8%), seizures (*n* = 34 of 892, 3.8%), stroke (*n* = 22 of 892, 2.5%), agitation or irritability (*n* = 15 of 892, 1.7%), drowsiness (*n* = 14 of 892, 1.6%), fatigue or lethargy (*n* = 13 of 892, 1.5%), muscle weakness (*n* = 13 of 892, 1.5%), hallucinations (*n* = 11 of 892, 1.2%), cerebellar ataxia (*n* = 10 of 892, 1.1%), gait instability or difficulty walking (*n* = 9 of 892, 1.0%), and dysarthria (*n* = 3 of 892, 0.3%).

Drowsiness was found to be more prevalent among children (*n* = 8 of 78, 7.3%) as opposed to adolescents (*n* = 1 of 52, 1.3%). More children also experienced muscle weakness (*n* = 8 of 78, 7.3%) in comparison to adolescents (*n* = 5 of 52, 6.3%). Lastly, there was a higher frequency of agitation or irritability among children (*n* = 8 of 78, 7.3%) than infants (*n* = 1 of 6, 1.3%).

Neurological impairment in the pediatric population involved splenial lesions of the corpus callosum (*n* = 29 of 892, 3.3%). Brain regions affected included the brainstem (*n* = 9 of 892, 1.0%), olfactory gyrus (*n* = 8 of 892, 0.9%), cerebellum (*n* = 7 of 892, 0.8%), temporal lobe (*n* = 6 of 892, 0.7%), frontal lobe (*n* = 5 of 892,0.6%), hippocampus (*n* = 2 of 892, 0.2%), parieto-occipital cortical (*n* = 2 of 892, 0.2%), frontal-parietal cortical (*n* = 1 of 892, 0.1%), midbrain (*n* = 1 of 892, 0.1%), and cerebrum (*n* = 1 of 892, 0.1%). Moreover, patients experienced CNS involvement (*n* = 54 of 892, 6.1%), PNS involvement (*n* = 31 of 892, 3.5%), or combined CNS-PNS involvement (*n* = 7 of 892, 0.8%).

Other neurological abnormalities consequent to COVID-19 positivity included MIS-C (*n* = 216 of 892, 24.2%); neuroinflammation (*n* = 90 of 892, 10.1%), including ADEM or encephalitis or myelitis or meningism or meningitis or meningoencephalitis; encephalopathy (*n* = 72 of 892, 8.1%); Guillain-Barre Syndrome (*n* = 25 of 892, 2.8%); Middle East Respiratory Syndrome (MERS; *n* = 10 of 892, 1.1%); infarct (*n* = 7 of 892, 0.8%); cerebral edema (*n* = 5 of 892, 0.6%); and vasculitis (*n* = 2 of 892, 0.2%). It should be noted that abnormalities were identified using the following imaging modalities: MRI (*n* = 263 of 358, 73.5%); CT (*n* = 78 of 358, 21.8%); CT and MRI (*n* = 10 of 358, 2.8%); and PET (*n* = 7 of 358, 2.0%).

## Discussion

Although children present with milder symptoms of COVID-19, 12.8% of COVID-19 positive, hospitalized pediatric patients had experienced neurocognitive impairments across the 19 included studies. Specifically, 24.2% of patients with neurological abnormalities had MIS-C, which was found to be the most prevalent manifestation, followed by neuroinflammation (10.1%) —including ADEM or encephalitis or myelitis or meningism or meningitis or meningoencephalitis—and encephalopathy (8.1%). When the data was categorized according to age groups, we observed that drowsiness and muscle weakness were more common among children than adolescents, and that agitation or irritability was more common among children than infants.

Many mechanistic theories have been proposed to gain a better understanding of the possible associations between COVID-19 and neurological manifestations in the pediatric population. The SARS-CoV-2 virus is known to have neurotropic abilities, using the ACE2 receptor as an entry way into host cells (17–20). It is suggested that the resulting disruption of intracellular neural homeostasis may lead to inflammation and disruption of the blood-brain barrier. This overarching proinflammatory phenomenon may explain why headaches and seizures were among the most commonly reported neurological symptoms—as well as in our findings (16.8% and 3.8%, respectively) (21). A possible explanation involves the direct entry of SARS-CoV-2 through ACE2 and/or the olfactory tract (22–24). The protective blood-brain barrier becomes disrupted, resulting in endotheliopathy and an immunologically-directed assault on the CNS (25, 26). As a result, the immune system becomes exposed to novel CNS antigens (27, 28). An alternative hypothesis states that there is an elevation of systemic inflammatory markers post-viral infection, causing neurological symptoms representing injury consequent to activation of the systemic autoinflammatory system (27, 29, 30).

Recently, multiple studies have reported a growing number of COVID-19-infected children developing this systemic inflammatory illness—now formally recognized as MIS-C (31–34). It is theorized that MIS-C may manifest as part of a post-infectious immune response – similar to the mechanism involved in the COVID-19-related autoimmune meningoencephalitis observed in adults (35). Furthermore, the MIS-C-induced cytokine storm has been proposed as another probable cause of the neurologic manifestations.

Evidence is also emerging on a potential association between COVID-19 and demyelinating disorders, such as Guillain-Barré Syndrome (GBS), in post-infectious adolescents (36–38). It is hypothesized that COVID-19 can achieve molecular mimicry due to similar antigenic factors to neurons (38–41). Such similarity enables interaction between COVID-19 and myelin autoantigens – ultimately causing myelin and neurologic damage, as seen in GBS (42, 43). As syndromes such as MIS-C and GBS are relatively uncommon and understudied, longitudinal studies are needed to gain greater insight into their neuropathophysiological mechanisms and association with COVID-19—especially among different age groups within the pediatric population: infants, children, and young adults.

Despite numerous studies reporting on the neurological manifestations of COVID-19 in adults, literature about the impact of COVID-19 on the brain structure and function of children and adolescents still remains limited. In the early stages of the COVID-19 pandemic, it appeared that the pediatric population was significantly less affected by the virus than adults were – where most were either asymptomatic, pre-symptomatic, or had mild symptoms (3). Although a number of explanations were proposed for the decreased impact of SARS-CoV-2 infection in children, there is still no significant evidence to support these claims (42). As the pandemic progressed, cases of children becoming seriously ill surfaced and became more widespread—among which some children acquired MIS-C requiring intensive care and the less severe, Kawasaki-like disease (27, 44–47).

When comparing adult cases to pediatric cases, it was found that most adult patients did not exhibit the respiratory symptoms of the initial COVID-19 infection (35). Additionally, Monrand et al. conducted a case series of seven adolescents who displayed persistent functional complaints following a confirmed or suspected SARS-CoV-2 infection. They showed evidence of a PET hypometabolism pattern involving the olfactory gyrus and medial temporal lobes that extended to the pons and cerebellum—similar to that previously discovered in adults with long COVID (48). This data supports the possibility of long COVID in children, given that all participants—regardless of age—shared a functional brain involvement during the acute COVID-19 stage (48, 49). However, it is important to highlight that there is limited knowledge on the neurological involvement and long-term effects of the SARS-COV-2 virus on children and young adults, as there is currently no substantial research examining the subject (50).

Future research must urgently focus on identifying subsets of children who are more susceptible to developing the aforementioned autoimmune manifestations – particularly as more children are returning to school and the risk of transmission heightens. It will also be important to investigate the impact of vaccination status in preventing these neurological presentations. Additional longitudinal studies are needed to establish a stronger relationship between pediatric neurological impairment and SARS-CoV-2 infection as an underlying etiological factor, thoroughly chart the neurological presentations of COVID-19 into groups of syndromes based on observed patterns, investigate pediatric specific cohorts at presentation along with long-term follow-up, and ascertain underlying mechanisms as well as promising therapeutic interventions. The foregoing neurological aspects associated with COVID-19 overlap with some observations in adults similarly infected with COVID-19, and add to the previously observed elevated rate of neurological and psychiatric disorders consequent to the pandemic (51–53).

### Limitations

The study has certain limitations. A key limitation of this study was the primary use of case reports/series and retrospective studies. Our data showed characteristic imaging abnormalities in children with MIS-C associated with COVID-19, but its frequency among all afflicted children is uncertain. Additionally, only hospital-recognized cases of COVID-19-related neurological involvement were included, which may not adequately reflect the scope and severity among the entire pediatric population. Another limitation was the lack of data available on the longitudinality of neurological presentations. Finally, specific neurological complications—such as olfactory impairment, which was commonly reported in adults—should also be studied in the pediatric population by functional neuroimaging study (54).

## Conclusion

This systematic review evaluates the association of SARS-CoV-2 infection with neurological symptoms and neuroimaging manifestations in the pediatric population and shows that, while a significant percentage of children and adolescents experienced MIS-C, neuroinflammation, and encephalopathy were—by comparison—moderately common. However, due to limited data on pediatric-specific subsets, more longitudinal studies are required to characterize the heterogenous neurological presentations of COVID-19 into either ‘infant,’ ‘children’, or ‘adolescent’ categories. Hence, with loosening pandemic restrictions, our focus must urgently address this gap in knowledge in order to identify the demographic that is most vulnerable to developing COVID-19-associated autoimmune disorders.

## Figures and Tables

**Table 1:** Quality assessment of included studies (*n* = 19).

Study Name	Study JBI score	Max JBI score	Applied JBI Tool
Abdel-Mannan et al. 2020	7	10	Case Series
Aljomah et al. 2021	5	10	Case Series
Dilber et al. 2021	8	8	Cross-sectional Studies
Dominguez et al. 2021	8	8	Cross-sectional Studies
Fenlon III et al. 2021	8	8	Cross-sectional Studies
Hatipoglu et al. 2020	6	8	Case Reports
Khan et al. 2021	7	10	Case Series
Krueger et al. 2021	6	10	Case Series
LaRovere et al. 2021	8	8	Cross-sectional Studies
Lindan et al. 2021	7	10	Case Series
Mihai et al. 2022	7	8	Cross-sectional Studies
Monrand et al. 2021	8	10	Case Series
Olivotto et al. 2021	7	10	Case Series
Orman et al. 2021	7	8	Cross-sectional Studies
Palabikik et al. 2021	8	8	Cross-sectional Studies
Ray et al. 2021	8	11	Cohort Studies
Sa et al. 2021	6	8	Cross-sectional Studies
Sandoval et al. 2021	8	10	Case Series
Ucan et al. 2022	9	10	Case Series

**Table 2:** Data summaries of included papers (*n* = 19).

Study	Country	Study Design	Data Acquisition Period	Total Sample Size (COVID-19 positive)	Number of Participants with Neurological Symptoms & Involvement	Sample Characteristics of Participants with Neurological Involvement	Mode(s) of Ascertainment	Summary of Findings
Abdel-Mannan et al., 2020	United Kingdom	Case Series	March 1, 2020 to May 8, 2020	*N* = 50 • 27 with Pediatric Multisystem Inflammatory Syndrome (MIS) • Sex (%F/%M): 50 (*n* = 25) /50 (*n* = 25)	*N* = 4	• Age Range: < 18 years • Median Age: 12 (range, 8–15) years	**• COVID-19**: QRT-PCR *(Nasopharyngeal Swab)* or SARS-CoV-2 IgG *(Serology)* • **Neurological Changes**: MRI	• Neurological Involvement: 8% (*n* = 4) * MIS: 14.8% (*n* = 4 of 27) - Brainstem signs with dysarthria or dysphagia (n = 2), cerebellar ataxia (n = 1), encephalopathy (n = 4), headache (n = 3), and meningism (n = 1) - Peripheral nervous system (PNS) involvement with global proximal muscle weakness (n = 4) and reduced reflexes (n = 2) − 45.1% (*n* = 23 of 50 pediatric and adolescent patients experienced neurological symptoms related to cytokine storms • **Brain CT/MRI Findings**: * Signal changes in the splenium of the corpus callosum (*n* = 4) - **Patient 1 (CT scan)**: hypodensity of the splenium of the corpus callosum - **Patient 2 (MRI)**: signal changes of the genu and SCC & bilateral centrum semiovale with restricted diffusion - **Patient 3 (MRI)**: hyperintensities with restricted diffusion in the SCC and bilateral centrum semiovale - **Patient 4 (MRI)**: signal changes in SCC with mild restricted diffusion
Aljomah et al., 2021	Saudi Arabia	Case Series	May 23, 2020 to June 30, 2020	*N* = 6	*N* = 5	• Age Range: Newborn-10 years • Sex: 1 female	**• COVID-19**: QRT-PCR *(Nasopharyngeal Swab)* • **Neurological Changes**: CSF analysis, EEG, and Brain & Spine MRI	• Neurological Involvement: 83% (*n* = 5) * **Case 1**: dysarthria and gait instability followed by double vision and ophthalmoplegia (developed Miller Fisher syndrome) * **Case 2**: fever and seizure * **Case 3**: headache and papilledema * Case 4: abnormal MRI findings (and congenital heart disease) * Case 5: history of abnormal movement post-sleep Note that **cases 4 and 5** had neurological findings not necessarily related to the virus that was discovered by incidence due to active screening. • **Neuroimaging Findings**: Spine MRI * Case 1: thickening and enhancement of the nerve roots of the cauda equina and conus medullaris, with no acute intracranial pathology Brain MRI * **Case 4**: rotated vermis and hypoplastic corpus callosum
Dilber et al., 2021	Turkey	Retrospective Observational	March 11, 2020 to January 30, 2021	*N* = 382 • Sex (%F/%M): 49.2 (*n* = 188)/50.8 (*n* = 194)	*N* = 259	• Mean Age: 7.14 ± 5.84 years • Median Age: (range, 0–17) years	**• COVID-19**: QRT-PCR *(Nasopharyngeal Swab)*, either with a positive result for IgG or IgM antibodies against SARS-CoV-2	• **Neurological Involvement: 68% (*n* = 259)** Hospitalization: 8.9% (*n* = 34) * Seizure: 52.9% (*n* = 18) * Headache: 38.2% (*n* = 13) * Dizziness: 8.8% (*n* = 3) * Meningoencephalitis: 5.8% (*n* = 2) * Increased frequency of seizures: 1.3% (*n* = 5) Outpatient: 58.9% (*n* = 225) * Headache: 23% (*n* = 88) * Dizziness: 14.3% (*n* = 55) * Anosmia: 10.2% (*n* = 39) * Ageusia/dysgeusia: 7.3% (*n* = 28) • **Cranial MRI Findings**: * Case 1: meningoencephalitis * Case 2: meningoencephalitis * **Severely symptomatic children who received inpatient care**: normal
Caro-Domínguez et al., 2021	France, Iran, Mexico, Peru, Spain, and Switzerland	Retrospective Observational	May 26, 2020 to June 21, 2020	*N* = 37	*N* = 37 • 12 (32%) patients with Pediatric MIS needed brain imaging (9 MRI and 3 CT)	• Age Range: 3 months-15.8 years • Sex (%F/%M): 43 (*n* = 16) /56 (*n* = 21)	**• COVID-19**: PCR Test in 15/37 (41%) or SARS-CoV-2 IgG *(Serology)* in 13/19 (68%) • **Neurological Changes**: CT (3/12) and MRI (9/12)	• **Neurological Involvement**: * MIS: 100% (*n* = 37) * Fever (*n* = 37, 100%), abdominal pain (*n* = 25, 68%), conjunctivitis (*n* = 14, 38%), cough (*n* = 12, 32%), and rash (*n* = 20, 54%) • **Brain Imaging Findings (*n* = 12)**: * Abnormal findings: 25% (*n* = 3 of 12) * Leptomeningeal enhancement of the right precentral sulcus (*n* = 1) * Foci of restricted diffusion in the splenium of the corpus callosum (*n* = 1) * Generalized atrophy of the cerebrum with decreased volume and abnormal signal of the white matter (*n* = 1) - Most likely related to her cardiac disease rather than COVID-19
Fenlon III et al., 2021	United States	Retrospective Observational	April 1, 2020 to July 31, 2020	*N* = 39 (includes up to age 20) • Sex (%F/%M): 53 (*n* = 25)/47 (*n* = 22)	*N* = 4 patients (9%) with brain MRI	• Mean Age: 8.4 (range, 1.3–20) years	**• COVID-19**: RT-PCR *(Nasopharyngeal Swab)* or SARS-CoV-2 IgG *(Serology)*, or both	• **Brain MRI Findings**: * Parieto-occipital cortical abnormality, possible PRES (*n* = 1) * Papilledema (*n* = 1) * Normal (*n* = 2) * Headache (*n* = 1)
Hatipoglu et al., 2020	Turkey	Case Report	April 2020	*N* = 3	*N* = 3	• Age: 13, 13, and 13 years old • Sex: male, male, and female, respectively	• **COVID-19**: RT-PCR (Throat and Nasopharyngeal Saliva)	• Smell and/or taste disorder developed without nasal symptoms (nasal congestion, nasal obstruction, or rhinorrhea) • **Brain MRI Findings (*n* = 3)**: * Case 1: no signal change or contrast material uptake was observed in the olfactory bulb; headache * Case 2: no anomalies of the olfactory bulbs and tracts * Case 3: no pathological imaging finding in the olfactory region; headache; weakness
Khan et al, 2021	India	Case Series	N/A	*N* = 3	*N* = 3	• Age: 11, 7, and 15 years old • Sex: male, female, and female, respectively	• **COVID-19**: SARS-CoV-2 IgG *(Serology)*	• **Brain CE-MRI Findings**: * **Case 1**: acute cerebellitis; headache; seizure * **Case 2**: encephalomyelitis * **Case 3**: arterial ischemic stroke - Small hypodense area involving subcortical white matter in the right frontal lobe suggestive of infarct and a small intracerebral hematoma in left frontal lobe with mild perilesional edema; headache; seizure **Note**: on follow-up visits, no cases had any neurological deficits
Krueger et al., 2021	Brazil	Case Series	N/A	*N* = 4	*N* = 4	• Age: 16 years, 15 years, 5 years, 2 months old • Sex: female, male, female, male, respectively	• **COVID-19**: RT-qPCR	• **Case 1**: * **Brain MRI Findings**: contrast enhancement in the anterior roots of the medullary cone and bilateral cranial * Sensory and motor polyradiculopathy with RT-qPCR for COVID-19 and dengue both detected in CSF that improved after appropriate treatment • **Case 2**: * Normal brain MRI findings * Guillain-Barre syndrome that improved after using human immunoglobulin * Weakness • **Case 3**: * Normal brain MRI findings * Acute intracranial hypertension that improved with lumbar puncture and using acetazolamide * Headache • **Case 4**: * Normal brain MRI findings * Focal epileptic seizures that recovered after antiepileptic treatment
LaRovere et al., 2021	United States	Retrospective Observational	March 15, 2020 to December 15, 2020	*N* = 1695 • Sex (%F/%M): 46 (*n* = 786)/54 (*n* = 909)	*N* = 365	• Age Range: < 21 years • Median Age: 9.1 (interquartile range, 2.4–15.3) years	• **COVID-19**: RT-PCR and symptoms associated with acute COVID-19 or met US Centers for Disease Control and Prevention criteria for MIS-C	• Neurological Involvement: 22% (*n* = 365) * Patients with underlying neurologic disorders experiencing neurological involvement (*n* = 81 of 365, 22%) * Patients without underlying neurologic disorders experiencing neurological involvement (*n* = 113 of 1330, 8%) * Patients who were previously healthy (53%, *n* = 195 vs 54%, *n* = 723) and met criteria for MIS (35%, *n* = 126 vs 37%, *n* = 490) * Patients experiencing neurologic involvement: − 88% (*n* = 322) had transient symptoms and survived − 12% (*n* = 43) had life-threatening conditions associated with COVID-19: • Severe encephalopathy (*n* = 15; 5 with splenial lesions) • Stroke (*n* = 12) • ADEM (*n* = 8) • Guillain-Barre syndrome/variants (*n* = 4) • Acute motor-sensory axonal neuropathy (*n* = 1) • Acute fulminant cerebral edema (*n* = 4) **• Head CT performed (*n* = 63)** • **Brain MRI Findings (*n* = 54)**: * MIS-C: Patients who were previously healthy with diffuse abnormal T2 hyperintensities and reduced diffusivity involving the white matter and genu/splenium of the corpus callosum (*n* = 5) - Severe encephalopathy, focal neurologic deficits, and visual hallucinations (*n* = 4 of 5) * Meningoencephalitis (ADEM-like) (*n* = 1) * Acute arterial ischemic stroke (*n* = 1) * Acute hemorrhagic stroke (*n* = 1) * Guillain-Barre syndrome (*n* = 1) * Acute fulminant cerebral edema (*n* = 1) * Encephalitis (*n* = 1) * Severe encephalopathy (*n* = 1) * Cerebral venous sinus thrombosis (*n* = 1)
Lindan et al., 2021	Argentina, Brazil, France, India, UK, US, Peru, and Saudi Arabia	Case Series	April 30, 2020 to Sept 8, 2020	*N* = 38	*N* = 38	• Sex (%F/%M): 45 (*n* = 17)/55 (*n* = 21)	• **COVID-19**: PCR test of upper respiratory tract, SARS-CoV-2 IgM *(Serology)*, and cerebrospinal fluid (CSF) analysis	• Neurological Involvement: 100% (*n* = 38) * Resembled an immune-mediated parainfectious pattern of disease involving the brain, spine, cranial nerves, and nerve roots: 65% (*n* = 13 of 20) patients in categories 1 and 2 *or* 74% (*n* = 28 of 38) patients in all categories • **Brain MRI Findings:** Category 1: Acute COVID-19: 32% (*n* = 12) * Autoimmune manifestations: 50% (*n* = 6) - ADEM-like imaging pattern: 33% (*n* = 4) - Neuritis: 17% (*n* = 2) * Aggressive myelitis: 8% (*n* = 1) * Small left frontal infarct: 8% (*n* = 1) * Acute encephalopathy and fever: 8% (*n* = 1) * Choroid plexitis and fulminant CNS tuberculosis with ventriculitis, hydrocephalus, and focal cerebral abscesses: 8% (*n* = 1) * Meningitis, inflammatory vasculitis, and multiple infarcts in the setting of sepsis: 8% (*n* = 1) Category 2: Asymptomatic Acute or Subacute COVID-19: 21% (*n* = 8) * ADEM-like brain changes and long-segment central cord myelitis: 25% (*n* = 2) * ADEM-like brain changes and anti-N-methyl-D-aspartate receptor (anti-NMDAR) autoimmune encephalitis: 13% (*n* = 1) * Long-segment central cord myelitis: 13% (*n* = 1) * Neuritis: 50% (*n* = 4) - ADEM-like changes and myelitis (*n* = 1 of 4) * Extensive superior sagittal sinus thrombosis with parasagittal venous infarcts: 13% (*n* = 1) Category 3: MIS-C: 29% (*n* = 11) * Splenial lesions of the corpus callosum in isolation or in combination with other brain abnormalities: 64% (*n* = 7) * ADEM-like brain changes: 64% (*n* = 7) * Cranial nerve enhancement: 18% (*n* = 2) * Cauda equina enhancement: 9% (*n* = 1) * Myelitis: 9% (*n* = 1) * Multiple punctate foci of susceptibility-induced signal drop-out in the brain, consistent with microthrombi: 9% (*n* = 1) * Enhancing myositis of the facial or neck musculature: 36% (*n* = 4) Category 4: Indeterminate: 18% (*n* = 7) * Neuritis: 71% (*n* = 4) * ADEM-like brain changes: 29% (*n* = 2) - Myelitis (*n* = 1 of 2) - Antimyelin oligodendrocyte glycoprotein (MOG) antibodies (*n* = 1 of 2) * Cerebellitis and cranial neuritis: 14% (*n* = 1) * Vasculitis and a midbrain infarct unrelated to a co-infection: 14% (*n* = 1)
Mihai et al., 2022	Romania	Retrospective, Observational, Descriptive Study	August 2020 and May 2021	*N* = 30	*N* = 14 (with MIS-C)	• Sex (%F/%M): 29 (*n* = 4)/71 (*n* = 10)	• **COVID-19**: nasopharyngeal swab	• Neurological Involvement: 46.7% (*n* = 14) * Headache (*n* = 11), ataxia (*n* = 5), photophobia (*n* = 5), difficulty walking (*n* = 4), meningism (*n* = 9), diplopia (*n* = 1), new appeared strabismus (*n* = 1), drowsiness (*n* = 7), lethargy (*n* = 5), alteration of consciousness (*n* = 6), skin hyperaesthesia (*n* = 6) • **Craniocerebral CT-MRI Findings (n = 1)**: * Demyelinating lesions (n = 1)
Monrand et al., 2021	France	Retrospective Case Series	February 25 and October 10, 2020	*N* = 661	*N* = 7	• Age Range: 10–13 years old • Mean Age: 12 • Sex (%F/%M): 86 (*n* = 6)/14 (*n* = 1)	• **COVID-19**: RT-PCR	• Neurological Involvement: 1.06% (*n* = 7) * Headache (*n* = 4) * Fatigue (*n* = 5) • **Brain [**^**18**^**F]-FDG PET Findings**: a similar brain hypometabolic pattern observed in adult long COVID-19 patients appeared 5 months later * **Patients 1, 3, 4, 5, 6, 7**: involved the bilateral medial temporal lobes (*n* = 6) * **Patients 1, 3, 4, 5, 6, 7**: brainstem (*n* = 6) * **All Patients**: cerebellum (*n* = 7) * **Patients 1, 3, 4, 5, 6**: involved the right olfactory gyrus after small volume correction (*n* = 5), with partial PET recovery (*n* = 2) at follow-up
Olivotto et et al., 2021	Italy	Retrospective Observational	October 1, 2020 to February 15, 2021	*N* = 34	*N* = 7 • 4 neuroimaging studies	• Age Range: 0–18 years old • Sex (%F/%M): 57 (*n* = 4)/43 (*n* = 3)	• **COVID-19**: Clinical or laboratory evidence of SARS-CoV-2 infection and abnormal neuroimaging findings (MRI or CT)	• **Neurological Involvement**: * **Patients 1–4**: severe phenotype - Severe irritability, mood deflection and drowsiness, variably associated with headache, meningism, photophobia, oculomotor apraxia, gait disorder, pain and slow, *“whiny”* and repetitive speech with reduced verbal output, and preserved comprehension **- Patient 3**: two generalized tonic-clonic seizures during fever * Patients 5–7: mild phenotype - Diffuse encephalopathy, namely mild irritability, drowsiness, mood deflection, and headache • **Brain MRI Findings (*n* = 4)**: * **Patients 1–4**: normal
Orman et al., 2021	United States	Retrospective Observational	March 18, 2020 to September 30, 2020	*N* = 3694 • 217 (6.5%) neuroimaging studies	*N* = 20 • 43 neuroimaging studies	• Age Range: 0–18 years old • Sex (Female/Male): 12:8 (n of females = 8; n of males = 12)	• **COVID-19**: PCR test or serum antibodies	• Systemic & Neurologic Involvement: 0.54% (*n* = 20) * Acute COVID-19: 10% (*n* = 2) * No signs of acute pathology: 90% (*n* = 18) • **Brain MRI Findings (*n* = 4)**: * **Patients 1–4**: normal
Palabikik et al., 2021	Turkey	Retrospective Observational	March 1, 2020 to March 31, 2021	*N* = 45	*N* = 45	• Age Range: 52 days–16 years old • Median Age: 7.68 years • Sex (%F/%M): 40 (*n* = 18)/60 (*n* = 27)	• **COVID-19**: RT-PCR test of upper respiratory tract and serology for SARS-CoV-2 antibodies using blood or CSF samples	**• Neurological Involvement**: * Reversible splenial lesion syndrome (RESLES): 13% (*n* = 6, most common) • **Brain MRI Findings (*n* = 4)**: * **Patient 1**: - Diffusion restriction in oval form in posterior part of body of corpus callosum in diffusion-weighted sequences * Patient 2: - ADEM-like signal changes without diffusion restriction in the cerebellar hemispheres, periaqueductal region, mesencephalon, bilateral hypothalamic region, bilateral thalamus, lentiform nucleus, caudate nucleus and deep white matter, and subcortical area - Cranial MRI performed 5 days after the seizure: PRES (new pathological signal changes in bilateral parietooccipital region and bilateral frontoparietal region) * **Patient 3**: - ADEM-like pathological signal changes in bilateral symmetrical deep and subcortical white matter - Necrosis in the parenchyma *(follow-up)* - Laminar necrosis and acute hemorrhagic necrotizing encephalomyelitis * **Patient 4**: muscle weakness diagnosed with Guillain-Barre syndrome (spinal MRI)
Ray et al., 2021	United Kingdom	Prospective Cohort Study	April 2, 2020 and Feb 1, 2021	*N* = 52	• *N* = 27 (52%) were classified into the COVID-19 neurology group • *N* = 25 (48%) were classified into the Pediatric Inflammatory Multisystem Syndrome (PIMS-TS) neurology group • Cerebral or spinal imaging: 92% (*n* = 48 of 52) patients * MRI (*n* = 46) * CT (*n* = 11) − 58% (*n* = 28) had abnormal scans	• Sex (%F/%M): 42 (*n* = 22)/58 (*n* = 30) **COVID-19 neurology group**: • Age Range: 1–16 years old • Mean Age: 9 **PIMS-TS neurology group**: • Age Range: 1–17 years old • Mean Age: 10	• **COVID-19**: positive PCR *(respiratory or spinal fluid samples)*, anti-SARS-CoV-2 IgG (*Serology*), or both	• **Neurological Involvement: 52% (*n* = 27)** COVID-19 Neurology Group (median age: 9 years, range: 1–16 years) * 52% (*n* = 14) had encephalopathy: - Encephalopathy associated with status epilepticus (*n* = 7): 3 had no pre-existing epilepsy - Encephalitis (*n* = 5) - Isolated encephalopathy (*n* = 2) * 48% (*n* = 13) presented with a recognized neuroimmune disorder: - GBS (*n* = 5) - ADEM (*n* = 5): 3 had myelin oligodendrocyte glycoprotein (MOG) antibodies - Other acute demyelinating syndromes (*n* = 3) - Autoimmune (limbic) encephalitis (*n* = 1) * PNS involvement occurred independently as a separate disorder (GBS in all 5 cases) (*n* = 10) * Acute psychosis: 7% (*n* = 2) * Chorea: 7% (*n* = 2) * Previous basal ganglia stroke and diagnosed with a transient ischemic attack (*n* = 1) PIMS-TS Neurology Group (median age: 10 years, range: 1–17 years) * 88% (*n* = 22) had encephalopathy * CNS signs: 24% (*n* = 6) - Ataxia (*n* = 3) - Hemiplegia associated with hemorrhagic stroke (*n* = 1) - Brainstem signs associated with ischemic stroke (*n* = 1) - Left hemiplegia associated with ADEM (*n* = 1) * ADEM with MOG antibodies: 4% (*n* = 1) * PNS involvement was part of the multisystem presentation: 40% (*n* = 10/25) * Seizures: 16% (*n* = 4) - Focal seizures (*n* = 3) (status epilepticus, *n* = 1) - Subtle motor seizures associated with ongoing subclinical ictal activity (*n* = 1) * Behavioral changes: 36% (*n* = 9) - Hallucinations (at presentation): 24% (*n* = 6) - Headache or meningism: 40% (*n* = 10) • **Cranial MRI Findings**: PIMS-TS Neurology Group: 74% (*n* = 17 of 23) patients had abnormal neuroimaging * Signal changes in the splenium of the corpus callosum consistent with MERS: 28% (*n* = 7) * Acute stroke (one ischemic involving the anterior and middle right cerebral artery & one intraparenchymal hemorrhage in the right frontal lobe): 8% (*n* = 2) * Bilateral hyperintensities within the claustra due to ADEM: 1% (*n* = 1) ***Image findings for 3 cases (n = 3)***: * Patient 1: ADEM * Patient 2: PIMS-TS, encephalopathy, and MERS * Patient 3: Guillain-Barre syndrome **COVID-19 Neurology Group**: 44% (*n* = 11 of 25) patients had abnormal neuroimaging * Diffuse T2 or fluid-attenuated inversion recovery (FLAIR) signal abnormalities of the cerebral white matter or deep gray matter consistent with ADEM (*n* = 4) * Abnormal T2 signal involving the hippocampi and cortical diffusion restriction due to limbic encephalitis (*n* = 1) * Abnormal T2 signal in the periventricular and infratentorial regions consistent with demyelination in a child with an acute demyelinating syndrome (clinically isolated syndrome) (*n* = 1) * Intraorbital segment of the right optic nerve consistent with optic neuritis (*n* = 1) * Splenium of the corpus callosum consistent with mild encephalopathy with reversible splenial lesion (MERS) (*n* = 1) * Myelitis (extensive intramedullary whole spinal cord abnormal T2 with pre-existing diagnosis of adrenal neuroblastoma) (*n* = 1) * Thickening and enhancement of the cauda equina nerve roots supportive of GBS (*n* = 2)
Sa et al., 2021	United Kingdom	Retrospective Observational (Cross-sectional)	March 1, 2020 to June 30, 2020	*N* = 75 • Sex (%F/%M): 33 (*n* = 25)/67 (*n* = 50)	*N* = 9 (with MIS-C)	• Median Age: 10 years • Interquartile Range: 7.9 years	• **COVID-19**: RT-PCR *(Nasopharyngeal swab)* or serology * Active infection: 33% (*n* = 3) patients * Recent infection (negative PCR and positive IgG serology): 33% (*n* = 3) patients	• Neurological Involvement: 12% (*n* = 9) * Extensive stroke: 22% (*n* = 2) * Splenial lesion: 11% (*n* = 1) • **Brain CT/MRI Findings (*n* = 4)**: * **Patient 6**: subtle cortical changes as a possible sequelae of hypoxic event * **Patient 7**: acute infarction * Patient 8: focal diffusion restriction involving the splenium of the corpus callosum and mild signal changes in hippocampal regions * Patient 9: intraparenchymal hemorrhage and infarction
Sandoval et al., 2021	Chile	Case Series	April 1, 2020 to July 14, 2020	*N* = 90	*N* = 13 with new-onset neurologic manifestations • 5 neuroimaging scans	• Age Range: 15 months–17 years old • Median Age: 6.5 years • Sex (%F/%M): 61.5 (*n* = 8)/ 38.5 (*n* = 5)	• **COVID-19**: qPCR assay from a nasopharyngeal swab or by positive serology	• Neurological Involvement: 14.4% (*n* = 13) * Predominant CNS symptoms: 38% (*n* = 5) * Predominant PNS symptoms: 54% (*n* = 7) * CNS & PNS symptoms: 54% (*n* = 7) * Neurologic symptoms appeared at different times in relation to the infection: - Concomitant: 30.8% (*n* = 4) - Onset a few weeks after the infection was no longer active: 69.2% (*n* = 9) - Neurologic symptoms as the only manifestation of the disease: 23% (*n* = 3) * Seizures: 23% (*n* = 3) * Agitation: 15.4% (*n* = 2) * Hallucinations: 7.7% (*n* = 1) * Headache: 54% (*n* = 7) * Generalized muscle weakness: 61.5% (*n* = 8) * Papilledema: 7.7% (*n* = 1) * Encephalopathy: 15.4% (*n* = 2) * MIS-C: 54% (*n* = 7) * GBS: 7.7% (*n* = 1) • **Brain MRI/CT Findings (*n* = 5)**: * Normal brain CT findings (*n* = 3) * Frontal hypodensity by CT (later confirmed as an unenhanced subcortical lesion by MRI) (*n* = 1) * Multifocal demyelinating lesions by brain and total-spine MRI (*n* = 1)
Ucan et al., 2022	Turkey	Retrospective Review	August 2020 and March 2021	*N* = 47 • Sex (%F/%M): 45 (*n* = 21)/55 (*n* = 26)	*N* = 3 (with MIS-C)	• Age Range: 25 months–15 years • Median Age: 8.6 years	• **COVID-19**: RT-PCR and serology	• Neurological Involvement: 6% (*n* = 3) * Lethargy, irritability, confusion, encephalopathy, and seizures • **Cranial MRI Findings (*n* = 3)**: * **Patient 1**: MIS with cerebellar ataxia, eventually improved - Restricted diffusion & T2 hyperintensity involving the corpus callosum splenium & diagnosed with mild encephalopathy with reversible splenial lesion (MERS) - Lesion in corpus callosum (disappeared in the follow-up cranial MRI) * **Patients 2–3**: normal
